# Baloxavir marboxil, a novel cap-dependent endonuclease inhibitor potently suppresses influenza virus replication and represents therapeutic effects in both immunocompetent and immunocompromised mouse models

**DOI:** 10.1371/journal.pone.0217307

**Published:** 2019-05-20

**Authors:** Keita Fukao, Yoshinori Ando, Takeshi Noshi, Mitsutaka Kitano, Takahiro Noda, Makoto Kawai, Ryu Yoshida, Akihiko Sato, Takao Shishido, Akira Naito

**Affiliations:** 1 Shionogi & Co., Ltd., Osaka, Japan; 2 Shionogi Techno Advance Research Co., Ltd., Osaka, Japan; Deutsches Primatenzentrum GmbH - Leibniz-Institut fur Primatenforschung, GERMANY

## Abstract

Baloxavir marboxil (BXM) is an orally available small molecule inhibitor of cap-dependent endonuclease (CEN), an essential enzyme in the initiation of mRNA synthesis of influenza viruses. In the present study, we evaluated the efficacy of BXM against influenza virus infection in mouse models. Single-day oral administration of BXM completely prevented mortality due to infection with influenza A and B virus in mice. Moreover, 5-day repeated administration of BXM was more effective for reducing mortality and body weight loss in mice infected with influenza A virus than oseltamivir phosphate (OSP), even when the treatment was delayed up to 96 hours post infection (p.i.). Notably, administration of BXM, starting at 72 hours p.i. led to significant decrease in virus titers of >2-log_10_ reduction compared to the vehicle control within 24 hours after administration. Virus reduction in the lung was significantly greater than that observed with OSP. In addition, profound and sustained reduction of virus titer was observed in the immunocompromised mouse model without emergence of variants possessing treatment-emergent amino acid substitutions in the target protein. In our immunocompetent and immunocompromised mouse models, delayed treatment with BXM resulted in rapid and potent reduction in infectious virus titer and prevention of signs of influenza infection, suggesting that BXM could extend the therapeutic window for patients with influenza virus infection regardless of the host immune status.

## Introduction

Influenza virus can rapidly spread in populations and are responsible for seasonal influenza epidemics around the world every year [1]. Influenza virus infection can lead to serious and fatal outcomes, especially in elderly or immunocompromised patients [2]. Although influenza vaccination represents the key option for preventing influenza virus infection and some strategies have been investigated to optimize immunogenicity by exploring new vaccines, vaccination doses, timing or adjuvants, its benefit in immunocompromised individuals is somewhat controversial [3, 4]. Additionally, vaccine mismatch has frequently occurred between the vaccine strain and the circulating strain [5]. Therefore, anti-influenza drugs play an important role in the control of influenza virus infections especially for patients with or at risk of severe infection and complications.

Currently, neuraminidase (NA) inhibitors are the most widely used class of anti-influenza drugs [6]. However, the emergence of influenza viruses resistant to NA inhibitors is an issue of concern [7]. In addition, previous clinical studies have indicated that NA inhibitors must be administered within 48 hours of the onset of symptoms [8]. This is difficult to do, because diagnosis is often delayed [9]. Thus, novel therapeutics that can extend the therapeutic window are needed if treatment is started from more than 48 hours after the onset of symptoms. Toward this aim, the recent availability of high-quality structural information of the influenza virus RNA polymerase complex [10] has led to the development of antiviral drugs that target the critical roles of the proteins involved in virus replication [11]. For example, pimodivir (JNJ-63523872 or VX-787), a novel PB2 inhibitor of influenza A virus, was found to provide protection from mortality in mice infected with influenza A virus when dosing was initiated at up to 96 hours p.i. [12].

Baloxavir marboxil (BXM) is an orally available small molecule inhibitor of cap-dependent endonuclease (CEN), an enzyme residing on the PA subunit of the influenza virus polymerase that mediates the cap-snatching process during viral mRNA biosynthesis [13–15]. BXM has been approved for clinical use in adults and adolescents in Japan and the United States. Baloxavir acid (BXA), the active form of the prodrug BXM [16], exhibited several times greater antiviral activity for type A virus than type B virus, but shows potent and broad-spectrum inhibitory activity against seasonal, avian, and swine influenza viruses *in vitro* [17]. In phase 3 clinical trials, BXM treatment significantly improved the time to alleviation of influenza symptoms compared with the placebo and also reduced infectious virus titer and the duration of virus shedding more rapidly than oseltamivir in otherwise healthy patients (CAPSTONE-1) and high-risk patients (CAPSTONE-2) [18, 19]. In non-clinical and clinical resistance analysis, PA/I38 substitutions have been identified as a major pathway for reduced BXA susceptibility [14, 17, 18], but its impact on the clinical and virologic effectiveness of BXM in patients with influenza remains to be investigated. We previously reported that BXM monotherapy or in combination with oseltamivir achieved significant reductions in virus titer and ameliorated signs of infection arising from a lethal dose of A/Puerto Rico (PR)/8/34 (H1N1) strain in a mouse model [20]. In addition, more recently, we reported the therapeutic efficacy of BXM against lethal infection with avian H7N9 virus [21]. However, the difference of the therapeutic effect of BXM due to starting time of treatment and its effect with delayed oral dosing in an immunosuppressed mouse model are still unknown. In the present study, we evaluated the efficacy of delayed dosing of BXM against infections of influenza virus in immunocompetent and immunosuppressed mouse models of infection.

## Materials and methods

### Compounds

BXM was synthesized at Shionogi & Co., Ltd. (Osaka, Japan) [16]. OSP was purchased from Sequoia Research Products (Oxford, UK). Suspensions of BXM and solution of OSP were prepared with 0.5% methylcellulose 400 solution (MC, FUJIFILM Wako Pure Chemical Corporation, Osaka, Japan). The administration dose was determined by body weight (1mL per 100 g body weight).

### Cells and viruses

Madin-Darby canine kidney (MDCK) cells were obtained from the European Collection of Cell Cultures. A/PR/8/34 and B/Hong Kong (HK)/5/72 strains of influenza virus were obtained from the American Type Culture Collection. For virus quantitation, serial dilutions of lung homogenates (both lungs homogenized with 2 mL of DPBS and antibiotics) were inoculated onto confluent MDCK cells as described previously [22]. The presence of cytopathic effects (CPE) was determined under a microscope and virus titers were calculated as log_10_ 50% tissue culture infectious dose (TCID_50_)/mL. When no CPE was observed at the lowest dilution, titers of unquantifiable virus were defined as 1.5 log_10_ TCID_50_/mL.

### Animals

Specific-pathogen-free 6-week-old female BALB/c mice (Charles River Laboratories Japan, Inc.) were used in the study. Body weights and survival were monitored once daily. The mice were immediately euthanized and regarded to be dead in the analysis for survival time when they had lost more than 30% of their body weight compared to their weight pre-infection and/or exhibited tremors or excessive hyposthenia according to humane endpoints. The numbers of mice that survived, euthanized according to humane endpoints, or died before reaching the humane endpoints are summarized in S1–S7 Tables. Upon virus inoculation, the mice were anesthetized by intramuscular administration at 100 μL of an anesthetic solution containing 0.03 mg/mL medetomidine hydrochloride, 0.4 mg/mL midazolam, and 0.5 mg/mL butorphanol tartrate in saline. All mouse studies were conducted under applicable laws and guidelines and with the approval of the Shionogi Animal Care and Use Committee (Approval number: S15038D, S15054D, S16047D, S16056D and S16105D).

### Antiviral study in mouse models

#### Effect of single-day administration of BXM in a lethal infection mouse model

BALB/c mice were inoculated intranasally with 100 μL of A/PR/8/34 at an infectious dose of 1.38 × 10^3^ (6.9 × 50% lethal dose in mice [MLD_50_]) or 4.42 × 10^4^ TCID_50_ (222 × MLD_50_), or B/HK/5/72 at an infectious dose of 3.30 × 10^5^ (88 × MLD_50_) or 1.98 × 10^6^ TCID_50_ (525 × MLD_50_). Mice were administered BXM (0.05, 0.5 or 5 mg/kg for A/PR/8/34; 0.5, 5 or 50 mg/kg for B/HK/5/72) orally twice daily (bid) for 1 day starting immediately after infection. Since dosing of 10 mg/kg/day of OSP for 5 days in mice is equivalent to the dosing of OSP in a clinical setting [23], mice were administered 5 mg/kg (humanized dose) or 50 mg/kg (10 times the high dose) of OSP bid for 5 days as a comparison. Control mice were treated with 0.5% MC bid for 1 day. Nine to ten mice were assigned to each group and examined once daily for survival over 14 or 21 days p.i.. To examine the effects of BXM on viral replication in mice, five mice in each group described above were euthanized, and their lungs were removed on day 1 p.i.. For the survival study in A/PR/8/34 (1.38 × 10^3^ TCID_50_)-infected mice, marked body weight loss and ruffled fur was observed in one mouse, which died at day 2 post infection in the group administered 5 mg/kg of BXM (bid for 1 day). No other mice including untreated mice showed such a rapid deterioration of their general condition at both infectious doses in this study. Therefore, this mouse was judged to be abnormal and excluded from the data analysis.

#### Effect of delayed administration of BXM in a lethal infection mouse model

Mice infected with 100 μL of A/PR/8/34 (1.38 × 10^3^ TCID_50_) were treated orally with BXM at a dose of 1.5 or 15 mg/kg bid for 5 days from 24, 48, 72, or 96 hours p.i.. OSP at a dose of 5 mg/kg was administered orally bid for 5 days. Control mice were treated with 0.5% MC bid for 5 days. Mice were examined once daily for survival and body weight through 28 days p.i.. None of the mice died from unexpected factors during the observation period.

To examine the effects of BXM on viral replication in mice when treatment of BXM was initiated at 72 hours p.i., eight mice in each group were euthanized, and their lungs were removed on days 1, 3, 4, 6, 8, and 10 p.i..

#### Effect of delayed administration of BXM in an immunocompromised mouse model

To examine the efficacy of delayed dosing of BXM in immunocompromised host, we used an immunosuppressed mouse model given cyclophosphamide (CP, Endoxan; Shionogi and Co., Ltd., Osaka, Japan), an antitumor drug which reduces the activity of NK cells and inhibits the proliferative responses of T and B cells [22, 24]. Mice were treated subcutaneously with CP once daily at 24 hours pre-virus exposure and for up to 9 days p.i.. The dose of CP was set at 0.2 mg/mouse (approximately 10 mg/kg) based on the preliminary result showing that virus shedding was prolonged in the CP-treated mice at this dose compared to the CP-untreated mice (S1 Fig). CP-treated mice infected with 100 μL of A/PR/8/34 (100 TCID_50_) were orally administered bid for 5 days with BXM at a dose of 1.5, 15 or 50 mg/kg, OSP at a dose of 5 or 50 mg/kg or 0.5% MC, beginning at 120 hours p.i.. To determine the virus titer in the lungs, five mice in each group were euthanized on days 5, 6, 7, 8, 9 and 10 p.i..

#### Sequence analysis of PA genes

Viral RNA was isolated from lung homogenates of infected mice using the MagNA Pure LC Total Nucleic Acid Isolation Kit. The PA region of influenza virus was amplified by nested RT-PCR. The primer sequences used are available upon request. Sanger sequence analysis of the PA region was performed by Viroclinics Biosciences B.V (Rotterdam, Netherlands).

### Statistical analysis

Differences in survival time after virus infection were analyzed by log rank test. Comparisons of the proportion of body weight at each time point to initial body weight were analyzed by Student’s t-test and the mixed model repeated measures for the analyses in the immunocompetent and immunocompromised mouse models, respectively. If a mouse died before the analysis time point, the proportion of the body weight of the mouse was regarded as 70%. One-way analysis of variance followed by pairwise comparisons and a linear model with unequal variance were applied for evaluating the virus titers in the lungs for the analysis with the immunocompetent and immunocompromised mouse models, respectively. The fixed-sequence procedure was used to adjust the multiplicity except for the comparison in virus titers of the single-day administration study. Statistical analysis was performed using the statistical analysis software, SAS version 9.2 for Windows (SAS Institute, Cary, NC). Two-sided adjusted *P* values below 0.05 were considered as statistically significant.

## Results

### Effects of single-day oral administration of BXM against lethal influenza virus infection

**Fig 1:**
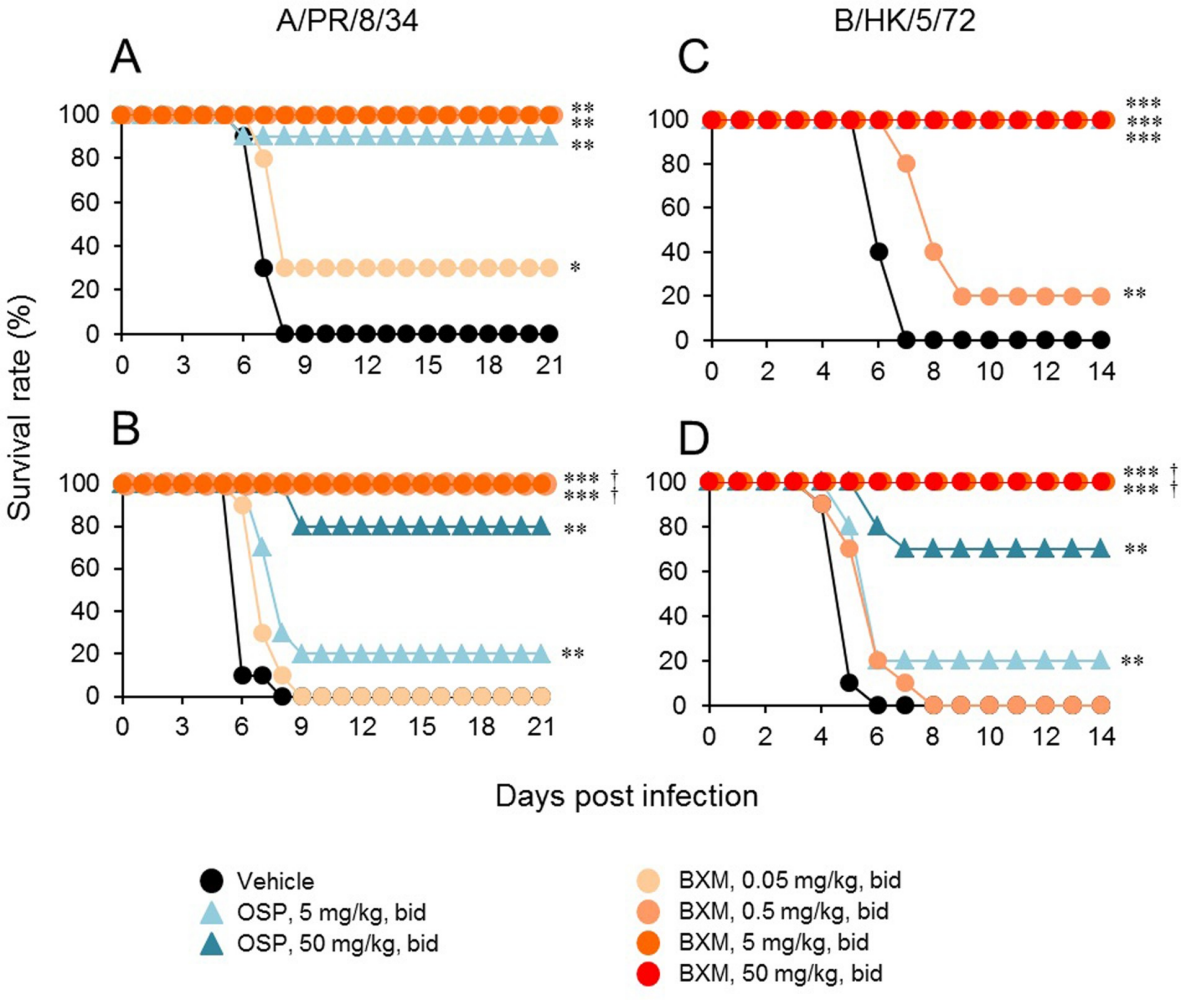
Therapeutic efficacy of single-day oral administration of BXM in a lethal infection mouse model. Nine to ten mice per group were intranasally infected with (A) 1.38 × 10^3^ or (B) 4.42 × 10^4^ TCID_50_ virus suspension of A/PR/8/34, or (C) 3.30 × 10^5^ or (D) 1.98 × 10^6^ TCID_50_ virus suspension of B/HK/5/72 and then orally administered BXM (0.05, 0.5, 5, or 50 mg/kg) bid for 1 day, OSP (5 or 50 mg/kg) bid for 5 days, or vehicle bid for 1 day. Mice were examined daily for survival through 14 or 21 days p.i.. The survival time in groups treated with BXM for 1 day was significantly prolonged compared to those in the vehicle-treated group (*, *P* < 0.05; **, *P* < 0.001; ***, *P* < 0.001) or OSP (5 mg/kg bid)-treated group (^†^, *P* < 0.0005).

All mice in the vehicle-treated group inoculated with A/PR/8/34 (1.38 × 10^3^ TCID_50_) died by day 8 p.i. (Fig 1A). Survival rates of groups treated with 0.05, 0.5, and 5 mg/kg bid of BXM were 30%, 100%, and 100%, respectively, while 90% of the mice treated with 5 mg/kg bid of OSP survived. All mice inoculated with A/PR/8/34 (4.42 × 10^4^ TCID_50_) without treatment also died by day 8 (Fig 1B). Survival rates of groups treated with 0.05, 0.5, and 5 mg/kg bid of BXM were 0%, 100%, and 100%, respectively, whereas only 20% of mice treated with 5 mg/kg bid of OSP survived. When mice were treated with 50 mg/kg bid of OSP, 80% survived. There were significant differences in survival time between groups treated with 0.5 or 5 mg/kg of BXM bid for 1 day and 5 mg/kg of OSP bid for 5 days. We observed a similar efficacy of BXM against lethal infection with B/HK/5/72 (Fig 1C and 1D). With consideration of the difference between antiviral activity of BXA against type A and that against type B virus *in vitro* [17], 10 times higher dose of BXM (0.5, 5, or 50 bid for 1 day) was administrated in the B/HK/5/72 infection model than A/PR/8/34 infection model. Single day administration of 5 or 50 mg/kg bid of BXM resulted in 100% survival in mice infected with 3.30 × 10^5^ TCID_50_ and 1.98 × 10^6^ TCID_50_ of B/HK/5/72. When the mice were administered 5 mg/kg bid of OSP, all mice infected with 3.30 × 10^5^ TCID_50_ survived, but for mice infected with 1.98 × 10^6^ TCID_50_, survival rates were 20% and 70% in the groups administered 5 and 50 mg/kg bid of OSP, respectively (Fig 1C and 1D).

**Fig 2:**
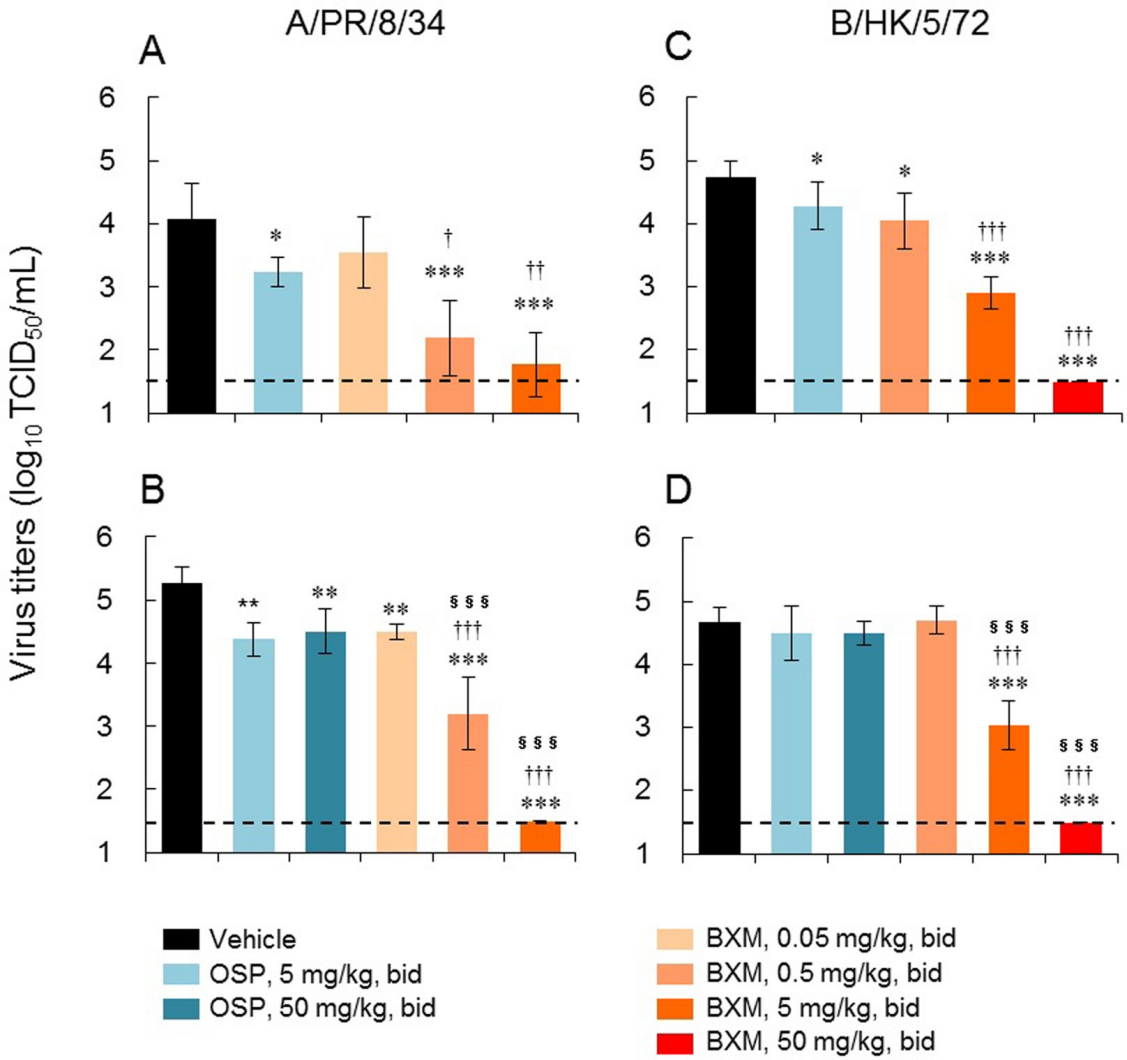
Inhibitory effects of single-day oral administration of BXM on virus titers in lungs in a lethal infection mouse model. Five mice per group were intranasally infected with (A) 1.38 × 10^3^ or (B) 4.42 × 10^4^ TCID_50_ virus suspension of A/PR/8/34, or (C) 3.30 × 10^5^ or (D) 1.98 × 10^6^ TCID_50_ virus suspension of B/HK/5/72 and then orally administered BXM (0.05, 0.5, 5, or 50 mg/kg), OSP (5 or 50 mg/kg), or vehicle bid for 1 day. The virus titers in lungs on day 1 p.i. were measured by TCID_50_ method. Each bar represents the mean ± SD of 5 mice. The limit of quantification (1.50 log_10_ TCID_50_/mL) is indicated by a dotted line. In the A/PR/8/34-infected mouse model, significant differences in virus titers were observed in BXM (except for 0.05 mg/kg bid at the infectious dose of 1.38 × 10^3^ TCID_50_)- and OSP-treated groups in comparison with the vehicle-treated group (*, *P* < 0.05; **, *P* < 0.001; ***, *P* < 0.0001). Significant differences in virus titers were also observed between BXM- and OSP-treated groups (^†^, *P* < 0.05; ^††^, *P* < 0.001; ^†††^, *P* < 0.0001 vs OSP 5 mg/kg bid; ^§§§^, *P* < 0.0001 vs OSP 50 mg/kg bid). In the B/HK/5/72-infected mouse model, significant differences in virus titers were observed in BXM (except for 0.5 mg/kg bid at the infectious dose of 1.98 × 10^6^ TCID_50_)- and OSP (at the infectious dose of 3.30 × 10^5^ TCID_50_)-treated groups in comparison with the vehicle-treated group (*, *P* < 0.05; **, *P* < 0.001; ***, *P* < 0.0001). Significant differences in virus titers were also observed between BXM- and OSP-treated groups (^†††^, *P* < 0.0001 vs OSP 5 mg/kg bid; ^§§§^, *P* < 0.0001 vs OSP 50 mg/kg bid).

In order to examine the inhibitory effect of BXM on virus replication in infected mice, the virus titers in the lungs on day 1 p.i. were determined. BXM treatment significantly and dose-dependently reduced virus titer at 24 hours after administration in mice lethally infected with influenza A or B virus (Fig 2). These reductions in virus titer were significantly greater than those observed with OSP.

### Effects of delayed administration of BXM against lethal influenza A virus infection in mice

**Fig 3:**
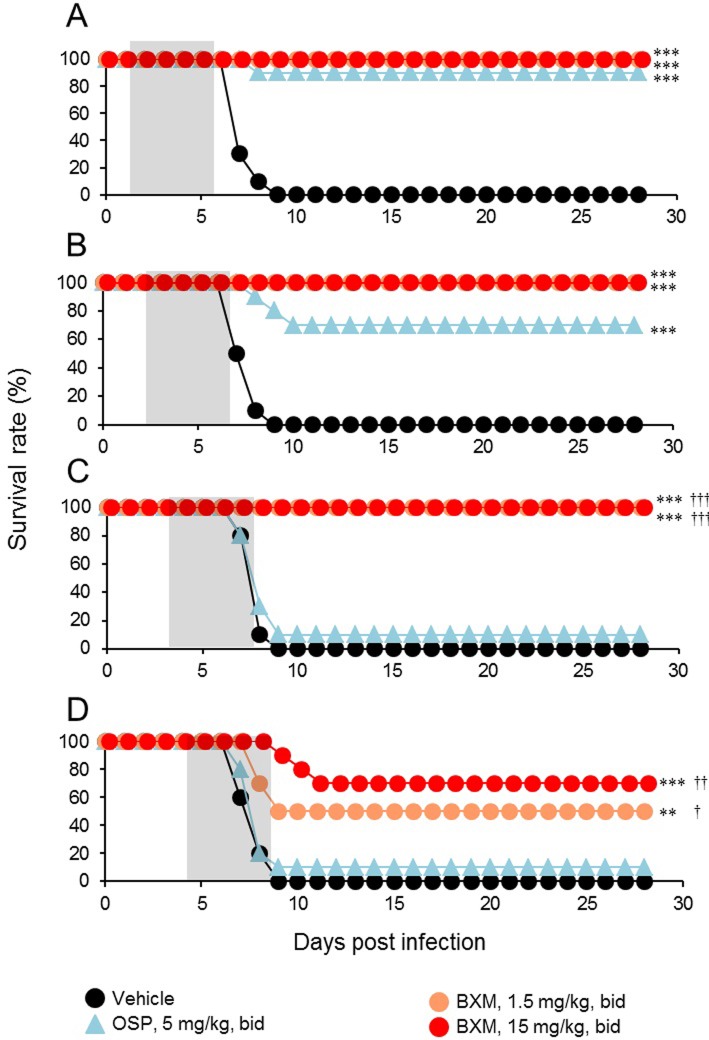
Therapeutic efficacy of delayed administration of BXM against lethal influenza A virus infection in mice. Ten mice per group infected with A/PR/8/34 (1.38 × 10^3^ TCID_50_) were treated orally with BXM (1.5 or 15 mg/kg), OSP (5 mg/kg), or vehicle bid for 5 days from (A) 24, (B) 48, (C)72, or (D) 96 hours p.i.. Mice were monitored daily for survival and body weight through 28 days p.i.. Treatment periods are indicated by the gray zones. Significant differences in survival time were observed in groups treated with BXM from 24, 48, 72, and 96 hours p.i. in comparison with the vehicle-treated group (**, *P* < 0.01; ***, *P* < 0.001). The survival time in groups treated with OSP from 24 and 48 hours p.i. was significantly prolonged compared to that in the vehicle-treated group (***, *P* < 0.0001). The survival time of the group that received BXM starting at 72 and 96 hours p.i. was significantly prolonged compared to that of the groups treated with OSP at a dose of 5 mg/kg bid (^†^, *P* < 0.05; ^††^, *P* < 0.01; ^†††^, *P* < 0.0001).

Next, we examined the effects of late administration of BXM in mice lethally infected with influenza A virus. Administration of 1.5 or 15 mg/kg bid of BXM was started from 24, 48, 72, or 96 hours p.i. and continued for 5 days. All vehicle-treated mice inoculated with A/PR/8/34 (1.38 × 10^3^ TCID_50_) died by day 9 p.i.. When treatment with BXM was delayed until 24, 48, or 72 hours p.i., all mice treated with 1.5 and 15 mg/kg bid of BXM survived (Fig 3A, 3B and 3C). Even when treatment of BXM was initiated at 96 hours p.i., survival rates of mice treated with 1.5 and 15 mg/kg bid of BXM were 50% and 70%, respectively (Fig 3D). In contrast, when OSP treatment was delayed until 24 or 48 hours p.i., the mice survived for a significantly longer period than the vehicle-treated group (Fig 3A and 3B), whereas the survival benefit decreased when treatment was started at 72 or 96 hours p.i. (Fig 3C and 3D). Comparing the efficacy of BXM and OSP on survival time for the same starting points of treatment, the survival times in the group given BXM at 72 and 96 hours p.i. were significantly prolonged compared with that of the OSP-treated group.

**Fig 4:**
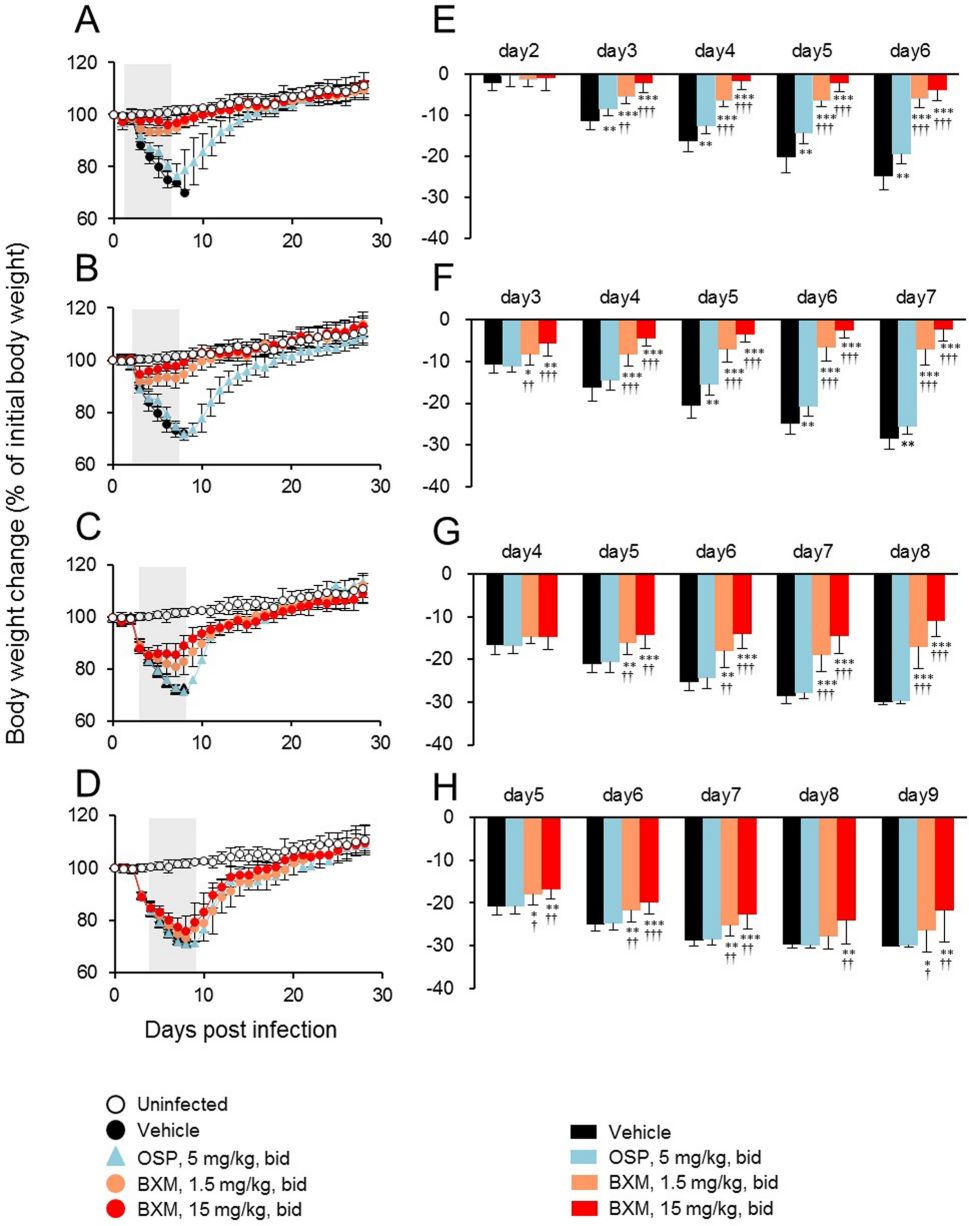
Effects of delayed administration of BXM on body weight change following influenza virus infection in mice. Mice infected with A/PR/8/34 (1.38 × 10^3^ TCID_50_) were orally treated with BXM (1.5 or 15 mg/kg), OSP (5 mg/kg), or vehicle bid for 5 days from (A, E) 24, (B, F) 48, (C, G) 72, or (D, H) 96 hours p.i. and monitored daily for body weight up to 28 days p.i.. Uninfected mice (n = 5) were also monitored daily for body weight as a control. Significant differences in body weight were observed in groups treated with BXM and OSP in comparison with vehicle-treated group on the indicated days (*, *P* < 0.05; **, *P* < 0.01; ***, *P* < 0.0001). The groups treated with BXM from 24, 48, 72 or 96 hours p.i. showed significantly less body weight loss than the OSP-treated group on the indicated days (^†^, *P*<0.05; ^††^, *P* < 0.01, ^†††^, *P* < 0.0001).

To further characterize the effects of delayed treatment with BXM, we compared body weight change during the treatment period for the same starting points of treatment. All groups treated with BXM from 24, 48, 72, or 96 hours p.i. showed significantly less reduction of body weight compared with the vehicle-treated group (Fig 4). Significant inhibitions in body weight loss were also observed in the groups treated with OSP at 24 and 48 hours p.i. compared with the control group, while the groups treated with OSP at 72 and 96 hours p.i. showed body weight loss comparable to the vehicle-treated group. These results suggest that BXM expands the therapeutic window and provides superior therapeutic benefit compared with OSP in our mouse model.

### Inhibitory effects of delayed administration of BXM on virus replication in mice

**Fig 5:**
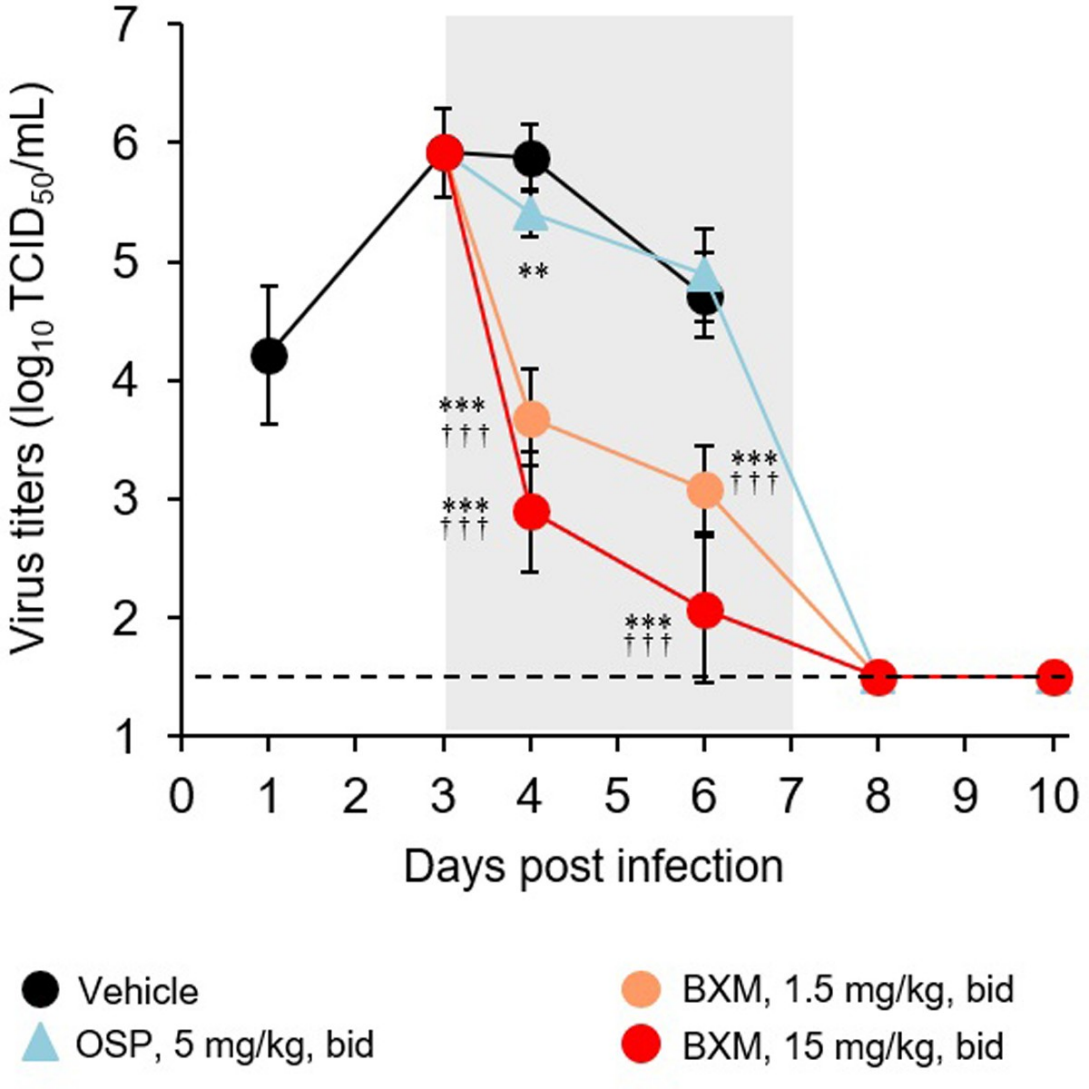
Inhibitory effects of delayed administration of BXM on virus replication in mice. Mice infected with A/PR/8/34 (1.38 × 10^3^ TCID_50_) were orally treated with BXM (1.5 or 15 mg/kg), OSP (5 mg/kg), or vehicle bid daily up to 5 days from 72 hours p.i. and were euthanized on the indicated days. Treatment period is indicated by the gray zone. Virus titers in the lungs were measured by the TCID_50_ method. The limit of quantification (1.50 log_10_ TCID_50_/mL) is indicated by a dotted line. Each point represents the mean ± SD of 6 to 8 mice except points that indicated virus titer on days 8 and 10 in mice treated with OSP (N = 1), in which only one mouse survived. Virus titers in mice treated with BXM and OSP on days 8 and 10 were at or lower than the quantification limit. Significant differences in virus titers were observed in BXM- and OSP-treated groups in comparison with the vehicle-treated group on the days 4 and 6, and day 4 p.i., respectively (**, *P* < 0.01; ***, *P* < 0.0001). Significant differences in virus titers were also observed between BXM- and OSP-treated groups on days 4 and 6 p.i. (^†††^, *P* < 0.0001).

To gain a better understanding of the mechanism by which delayed administration of BXM protects mice from a lethal virus inoculum, we examined the inhibitory effects on virus replication in the mouse lung. To do this, the mice inoculated with A/PR/8/34 (1.38 × 10^3^ TCID_50_) were administered 1.5 and 15 mg/kg of BXM bid daily up to 5 days starting at 72 hours p.i.. On days 4 and 6 p.i., virus titers in all groups treated with BXM were significantly reduced compared with those in the vehicle-treated group (Fig 5). Significant reductions in virus titer were observed in the group treated with 5 mg/kg bid of OSP compared with the vehicle-treated group on day 4 p.i., but not on day 6 p.i. Comparing the efficacy of BXM and OSP, the virus titer on days 4 and 6 p.i. in the groups treated with 1.5 and 15 mg/kg bid of BXM was significantly lower than that in the group treated with 5 mg/kg bid of OSP.

### Inhibitory effect of BXM on influenza virus replication in immunocompromised mice

**Fig 6:**
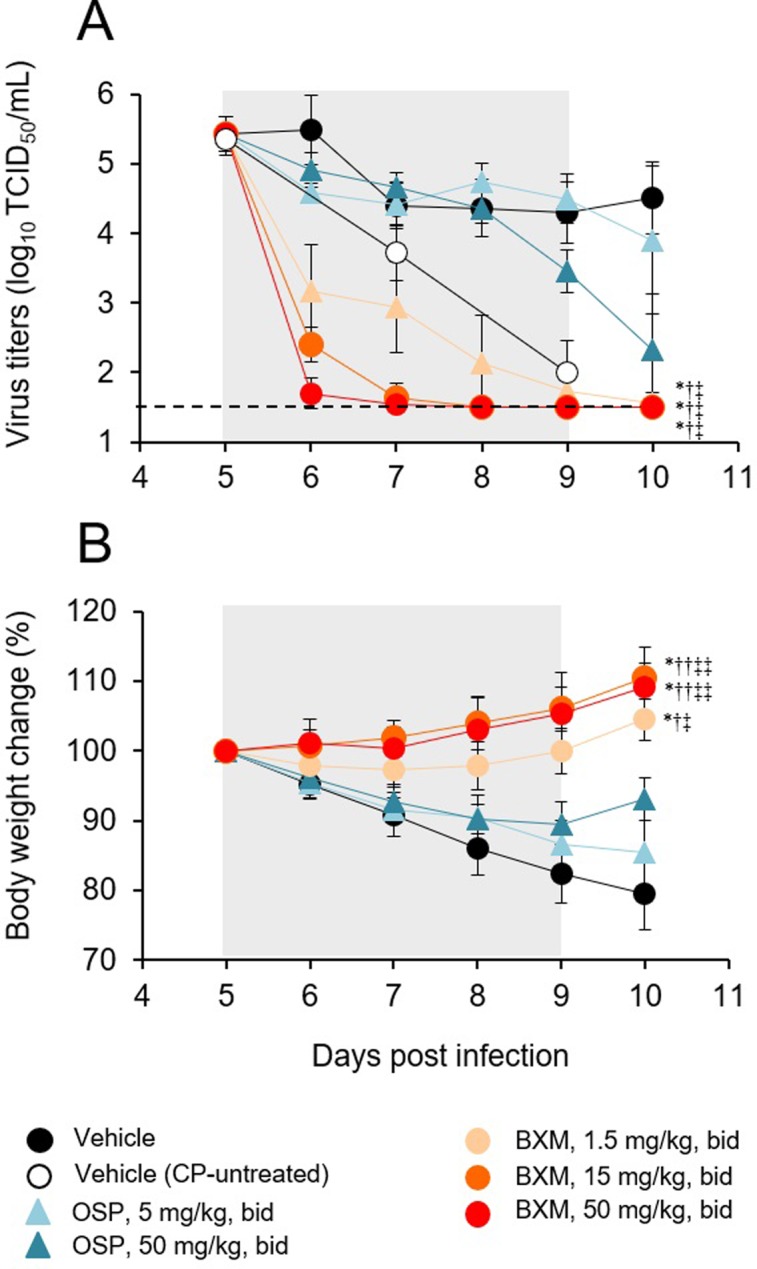
Effects of BXM on the virus titers and body weight changes in immunocompromised mice infected with influenza A virus. Five mice per group infected with A/PR/8/34 (100 TCID_50_) were treated orally with BXM (1.5, 15 or 50 mg/kg), OSP (5 or 50 mg/kg), or vehicle bid for 5 days from 120 hours p.i.. The mice were monitored daily for body weight up to day 10 p.i. and their lungs were harvested on days 5, 6, 7, 8, 9 and 10 p.i. to determine virus titers. Treatment periods are indicated by the gray zones. (A) A significant reduction in virus titer from days 5 to 10 p.i. was observed in all groups administered BXM in comparison with the group administered the vehicle or OSP at doses of 5 and 50 mg/kg bid (*, *P* < 0.0001 vs vehicle; ^†^, *P* < 0.0001 vs OSP 5 mg/kg bid; ^‡^, *P* < 0.0001 vs OSP 50 mg/kg bid). (B) All groups treated with BXM showed significantly less reduction of body weight compared with the vehicle-treated group or OSP-treated groups (*, *P* < 0.0001 vs vehicle; ^†^, *P* < 0.0005; ^††^, *P* < 0.0001 vs OSP 5 mg/kg bid; ^‡^, *P* < 0.005, ^‡‡^, *P* < 0.0001 vs OSP 50 mg/kg bid).

In order to evaluate the efficacy of repeated oral administration of BXM against influenza A virus infection in immunocompromised host, mice were treated with CP. Mice infected with A/PR/8/34 (100 TCID_50_) were orally administered 1.5, 15, or 50 mg/kg of BXM bid for 5 days, starting at 120 hours p.i., and then virus titers in the lungs were determined on days 5 to 10 p.i. While the virus titers in CP-untreated mice gradually decreased after day 5 p.i. (Fig 6A), the virus titers remained > 4 log_10_ TCID_50_/mL from day 5 to 10 p.i. in CP-treated mice, indicating that the period of virus infection was prolonged in immunosuppressed mice. Oral administration of BXM significantly and dose-dependently reduced virus titers within 24 hours after initial treatment, and the virus titers continued to gradually decrease until reaching the lower limit of quantification. In contrast, virus titers in OSP-treated groups remained at a similar level to those in the vehicle-treated group. In comparison to OSP-treated groups, the virus titers in groups treated with BXM remained significantly lower over 5 days after initiation of treatment. We also examined body weight changes for 5 days during the treatment period. The body weight loss was significantly suppressed even when treatment with 15 or 50 mg/kg bid of BXM was initiated at 120 hours p.i. (Fig 6B). In contrast, body weight loss in groups treated with 5 or 50 mg/kg bid of OSP-treated group was slightly but not significantly suppressed.

Finally, in order to monitor the emergence of variant viruses during BXM treatment in immunocompromised mice, we analyzed PA gene sequences of viruses obtained from lung homogenates on 6, 8, and 10 days p.i. by Sanger sequencing. No amino acid substitutions in the PA protein were detected in the viruses from the lung homogenates of mice treated with BXM when compared to the parent virus (S2–S4 Figs).

## Discussion

In this study, we demonstrated that oral administration of BXM was more effective than OSP for reducing mortality and virus titers and for ameliorating influenza symptoms in mice even when treatment was delayed, offering the basis for further investigation with both immunocompetent and immunocompromised patients.

In the single-day administration study, we investigated the dose-dependency of the effect of BXM treatment on survival and virus titers by evaluating the efficacy for a wide range of administration doses. We then examined whether oral administration of BXM could extend the therapeutic window against lethal infection with influenza A virus in mice. For this, we evaluated the efficacy of delayed treatment with BXM in mice at dose settings based on PK profiles in humans and mice. As we previously reported, from the results of pharmacokinetic/pharmacodynamic analysis, we consider that oral administration of 15 mg/kg of BXM bid for 5 days used in mice can mimic the plasma concentration of BXA in humans [18, 20, 25, 26]. Therefore, the efficacy of delayed treatment with BXM was evaluated at 15 mg/kg bid for 5 days in our mouse model to predict clinical effectiveness. Some higher and lower doses were also tested to confirm the dose-dependency. In this study, no toxicity issue was observed even in the high dose (50 mg/kg bid for 5 days) treatment group.

Currently, NA inhibitors are the most widely used class of anti-influenza drugs. They have been reported to ameliorate the symptoms of acute, uncomplicated influenza infection when treatment was started within 48 hours of the onset of the influenza symptoms [27]. However, some non-clinical and clinical studies have suggest that the antiviral efficacy of OSP is slightly limited [8, 11, 20]. Another previous non-clinical work evaluating the efficacy of delayed treatment with T-705, a broad spectrum inhibitor of RNA-dependent RNA polymerase, has also demonstrated that early initiation of treatment is strongly associated with a reduction of mortality in mice infected with a lethal inoculum of influenza virus [28]. In our immunocompetent lethal mouse model, OSP did not effectively reduce virus titers and virus-induced influenza signs. In contrast, delayed treatment with BXM was more effective for reducing the virus titers and preventing mortality and body weight loss than OSP. These findings suggested that BXM potently suppressed viral replication and prevented lethality when treatment was initiated not only in the early phase but also in the late phase of infection. Although delayed treatment with antivirals has been investigated and delayed treatment with pimodivir can improve survival in lethal mouse models of influenza [12], this agent has not yet been licensed for clinical use. The clinical significance of delayed treatment with BXM after 48 hours of onset of influenza symptoms needs to be further explored especially for patients with or at risk of severe infection and complications.

The theoretical benefit of delayed treatment with BXM was supported by its efficacy against influenza virus infection in immunocompromised mice. We confirmed that the immunocompromised mice showed prolonged replication of influenza virus from day 5 to day 10 p.i., which is similar to immunocompromised patients in which prolonged viral replication induced progressive illness and the emergence of antiviral resistance [2]. When treatment with BXM was initiated at 120 hours p.i., we found that BXM significantly reduced virus titers within 24 hours after initial treatment, and then significantly inhibited body weight loss in virus-infected immunocompromised mice, whereas OSP was not effective as previously reported in past studies using immunodeficient mouse model [29]. This finding shows that suppression of viral replication in the late phase of infection was crucial for ameliorating symptoms in our immunosuppressed mouse model.

Prolonged replication of influenza virus in patients given repeated treatments of an anti-viral drug might be a risk factor in the emergence of variants with reduced susceptibility [30, 31]. We have reported that I38 substitutions in PA were identified as markers for reduced susceptibility to BXA in patients treated with BXM [14], and the viruses containing these substitutions were identified in 2.2% (all with influenza A[H1N1]pdm09 infection) and 9.7% (all with influenza A[H3N2] infection) of the patients after treatment with BXM in phase 2 and phase 3 trials with otherwise healthy patients, respectively [18]. In the current study, no mutant virus with amino acid substitutions in PA was detected in immunocompromised mice during the treatment with BXM for 5 days (sampling on 6, 8, and 10 days p.i.), suggesting that BXM exerted its antiviral efficacy without the appearance of viruses possessing treatment-emergent amino acid substitutions during treatment in our immunocompromised mouse model. The emergence of NA inhibitor-resistant variants has been reported in nude and SCID mouse models with prolonged viral replication [32, 33], whereas no evidence of emergence of variant viruses was demonstrated in some studies with immunocompetent mouse models [34, 35]. Although virus shedding was prolonged by CP treatment as observed in immunocompromised patients, immunosuppression may be modest in our immunocompromised mouse model. Thus, the risk of emergence of variant viruses might be relatively low compared to the nude and SCID mouse models. In addition, it is suggested that I38 substitutions can emerge less frequently by influenza A (H1N1) infection compared with influenza A (H3N2) infection as observed in clinical studies described above [16]. Further clinical investigations are needed to shed light on the emergence of viruses with reduced susceptibility to BXA in immunocompromised patients.

## Conclusions

We demonstrated that oral administration of BXM had beneficial effects on survival, virus titers and signs of infection in not only immunocompetent but also immunocompromised mice infected with influenza A virus even when treatment was delayed until 4 or 5 days p.i., suggesting that treatment with BXM may extend the therapeutic window for patients with influenza virus infection.

## Supporting information

S1 Table The numbers of euthanized, died or survived mice in the study for examining the lethality in influenza A virus (A/PR/8/34)-infected mice. (DOCX)

S2 Table The numbers of euthanized, died or survived mice in the study examining the lethality in influenza B virus (B/Hong Kong/5/72)-infected mice. (DOCX)

S3 Table The numbers of euthanized, died or survived mice in the delayed administration study examining the lethality in influenza A virus (A/PR/8/34)-infected mice. (DOCX)

S4 Table The numbers of euthanized, died or survived mice in the study examining the viral titer in influenza A virus (A/PR/8/34)-infected mice. (DOCX)

S5 Table The numbers of euthanized, died or survived mice in the study examining the viral titer in influenza B virus (B/Hong Kong/5/72)-infected mice. (DOCX)

S6 Table The numbers of euthanized, died or survived mice in the delayed administration study examining the viral titer in influenza A virus (A/PR/8/34)-infected mice. (DOCX)

S7 Table The numbers of euthanized, died or survived mice in the study examining the viral titer in influenza A virus (A/PR/8/34)-infected immunocompromised mice. (DOCX)

S1 Fig The effect of cyclophosphamide on lung virus titers in mice infected with influenza A virus. BALB/c Mice were treated subcutaneously with CP (0 or 10 mg/kg) once daily at 24 hours pre-virus exposure and for up to 13 days p.i.. CP-treated mice were infected with 100 μL of A/PR/8/34 (100 TCID_50_). To determine the virus titer in lungs, 5 mice in each group were euthanized on days 8, 10, 12 and 14 p.i..
(TIF)

S2 Fig Amino acid sequence alignment of PA region of A/PR/8/34 strain (Day 6). Sanger sequence analysis of the PA region of A/PR/8/34 strain was performed. Sample RNA derived from vehicle-treated group (sampling on 5 days p.i.), treatment groups with BXM (sampling on 6 days p.i.), and the parent virus (A/PR/8/34 strain) were subject to this analysis. Dot plot indicates that the amino acid sequence of virus derived from the treatment group is identical to that of the parent virus.
(TIF)

S3 Fig Amino acid sequence alignment of PA region of A/PR/8/34 strain (Day 8). Sanger sequence analysis of the PA region of A/PR/8/34 strain was performed. Sample RNA derived from vehicle-treated group (sampling on 5 days p.i.), treatment groups with BXM (sampling on 8 days p.i.), and the parent virus (A/PR/8/34 strain) were subject to this analysis. Dot plot indicates that the amino acid sequence of virus derived from the treatment group is identical to that of the parent virus.
(TIF)

S4 Fig Amino acid sequence alignment of PA region of A/PR/8/34 strain (Day 10). Sanger sequence analysis of the PA region of A/PR/8/34 strain was performed. Sample RNA derived from vehicle-treated group (sampling on 5 days p.i.), treatment groups with BXM (sampling on 10 days p.i.), and the parent virus (A/PR/8/34 strain) were subject to this analysis. Dot plot indicates that the amino acid sequence of virus derived from the treatment group is identical to that of the parent virus.
(TIF)
