# Supplementary material for: Baloxavir marboxil, a novel cap-dependent endonuclease inhibitor potently suppresses influenza virus replication and represents therapeutic effects in both immunocompetent and immunocompromised mouse models
Source: PLoS One. 2019 May 20;14(5):e0217307. doi: 10.1371/journal.pone.0217307 (PMC6527232; doi:10.1371/journal.pone.0217307)
Supplement: S4 Table — (DOCX) [file pone.0217307.s004.docx]

| Infectious dose  (TCID_50_) | Group | Dosing period | Dose (mg/kg) | Euthanized^a^ / Died^b^ / Survived^c^ / Total per group | | | | | | |
| --- | --- | --- | --- | --- | --- | --- | --- | --- | --- | --- |
| 1.38 × 10^3^ | Vehicle | bid  for 1 day | – | 0 | / | 0 | / | 5 | / | 5 |
|  | OSP |  | 5 | 0 | / | 0 | / | 5 | / | 5 |
|  | BXM |  | 0.05 | 0 | / | 0 | / | 5 | / | 5 |
|  |  |  | 0.5 | 0 | / | 0 | / | 5 | / | 5 |
|  |  |  | 5 | 0 | / | 0 | / | 5 | / | 5 |
| 4.42 × 10^4^ | Vehicle |  | – | 0 | / | 0 | / | 5 | / | 5 |
|  | OSP |  | 5 | 0 | / | 0 | / | 5 | / | 5 |
|  |  |  | 50 | 0 | / | 0 | / | 5 | / | 5 |
|  | BXM |  | 0.05 | 0 | / | 0 | / | 5 | / | 5 |
|  |  |  | 0.5 | 0 | / | 0 | / | 5 | / | 5 |
|  |  |  | 5 | 0 | / | 0 | / | 5 | / | 5 |

a, euthanized according to humane endpoints; b, died before reaching humane endpoints; c, survived until the mouse was euthanized for lung sampling.
