# Supplementary material for: Baloxavir marboxil, a novel cap-dependent endonuclease inhibitor potently suppresses influenza virus replication and represents therapeutic effects in both immunocompetent and immunocompromised mouse models
Source: PLoS One. 2019 May 20;14(5):e0217307. doi: 10.1371/journal.pone.0217307 (PMC6527232; doi:10.1371/journal.pone.0217307)
Supplement: S7 Table — (DOCX) [file pone.0217307.s007.docx]

| Sampling time of lungs (hours post-infection) | Group | Dosing period | Dose (mg/kg) | Euthanized^a^ / Died^b^ / Survived^c^ / Total per group | | | | | | |
| --- | --- | --- | --- | --- | --- | --- | --- | --- | --- | --- |
| Untreated | Vehicle | – | – | 0 | / | 0 | / | 5 | / | 5 |
|  |  | bid for 2 days |  | 0 | / | 0 | / | 5 | / | 5 |
|  |  | bid for 4 days |  | 0 | / | 0 | / | 5 | / | 5 |
| Treated |  | – |  | 0 | / | 0 | / | 5 | / | 5 |
|  |  | bid for 1 day |  | 0 | / | 0 | / | 5 | / | 5 |
|  |  | bid for 2 days |  | 0 | / | 0 | / | 5 | / | 5 |
|  |  | bid for 3 days |  | 0 | / | 0 | / | 5 | / | 5 |
|  |  | bid for 4 days |  | 0 | / | 0 | / | 5 | / | 5 |
|  |  | bid for 5 days |  | 0 | / | 0 | / | 5 | / | 5 |
|  | OSP | bid for 1 day | 5 | 0 | / | 0 | / | 5 | / | 5 |
|  |  | bid for 2 days |  | 0 | / | 0 | / | 5 | / | 5 |
|  |  | bid for 3 days |  | 0 | / | 0 | / | 5 | / | 5 |
|  |  | bid for 4 days |  | 0 | / | 0 | / | 5 | / | 5 |
|  |  | bid for 5 days |  | 0 | / | 0 | / | 5 | / | 5 |
|  |  | bid for 1 day | 50 | 0 | / | 0 | / | 5 | / | 5 |
|  |  | bid for 2 days |  | 0 | / | 0 | / | 5 | / | 5 |
|  |  | bid for 3 days |  | 0 | / | 0 | / | 5 | / | 5 |
|  |  | bid for 4 days |  | 0 | / | 0 | / | 5 | / | 5 |
|  |  | bid for 5 days |  | 0 | / | 0 | / | 5 | / | 5 |
|  | BXM | bid for 1 day | 1.5 | 0 | / | 0 | / | 5 | / | 5 |
|  |  | bid for 2 days |  | 0 | / | 0 | / | 5 | / | 5 |
|  |  | bid for 3 days |  | 0 | / | 0 | / | 5 | / | 5 |
|  |  | bid for 4 days |  | 0 | / | 0 | / | 5 | / | 5 |
|  |  | bid for 5 days |  | 0 | / | 0 | / | 5 | / | 5 |
|  |  | bid for 1 day | 15 | 0 | / | 0 | / | 5 | / | 5 |
|  |  | bid for 2 days |  | 0 | / | 0 | / | 5 | / | 5 |
|  |  | bid for 3 days |  | 0 | / | 0 | / | 5 | / | 5 |
|  |  | bid for 4 days |  | 0 | / | 0 | / | 5 | / | 5 |
|  |  | bid for 5 days |  | 0 | / | 0 | / | 5 | / | 5 |
|  |  | bid for 1 day | 50 | 0 | / | 0 | / | 5 | / | 5 |
|  |  | bid for 2 days |  | 0 | / | 0 | / | 5 | / | 5 |
|  |  | bid for 3 days |  | 0 | / | 0 | / | 5 | / | 5 |
|  |  | bid for 4 days |  | 0 | / | 0 | / | 5 | / | 5 |
|  |  | bid for 5 days |  | 0 | / | 0 | / | 5 | / | 5 |

a, euthanized according to humane endpoints; b, died before reaching humane endpoints; c, survived until the mouse was euthanized for lung sampling.
