# Supplementary figures and images for: Baloxavir marboxil, a novel cap-dependent endonuclease inhibitor potently suppresses influenza virus replication and represents therapeutic effects in both immunocompetent and immunocompromised mouse models
Source: PLoS One. 2019 May 20;14(5):e0217307. doi: 10.1371/journal.pone.0217307 (PMC6527232; doi:10.1371/journal.pone.0217307)

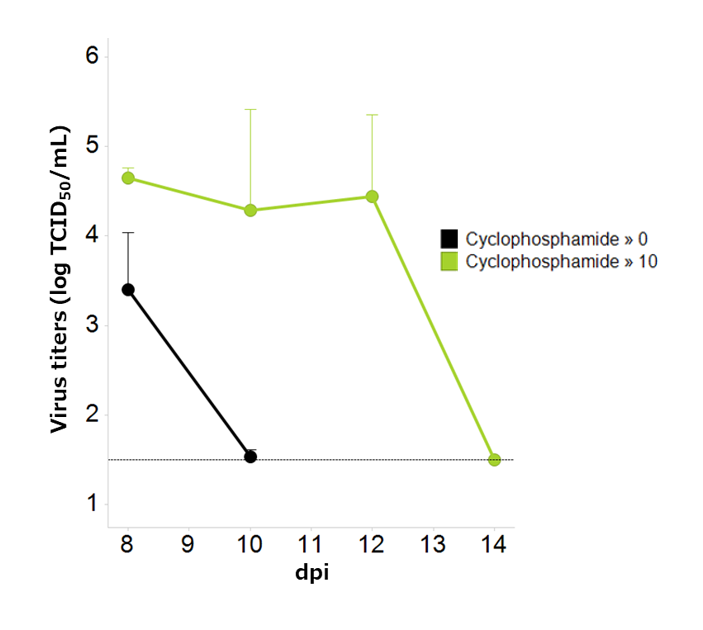

Supplement: S1 Fig — BALB/c Mice were treated subcutaneously with CP (0 or 10 mg/kg) once daily at 24 hours pre-virus exposure and for up to 13 days p.i.. CP-treated mice were infected with 100 μL of A/PR/8/34 (100 TCID50). To determine the virus titer in lungs, 5 mice in each group were euthanized on days 8, 10, 12 and 14 p.i.. (TIF) [file pone.0217307.s008.tif]

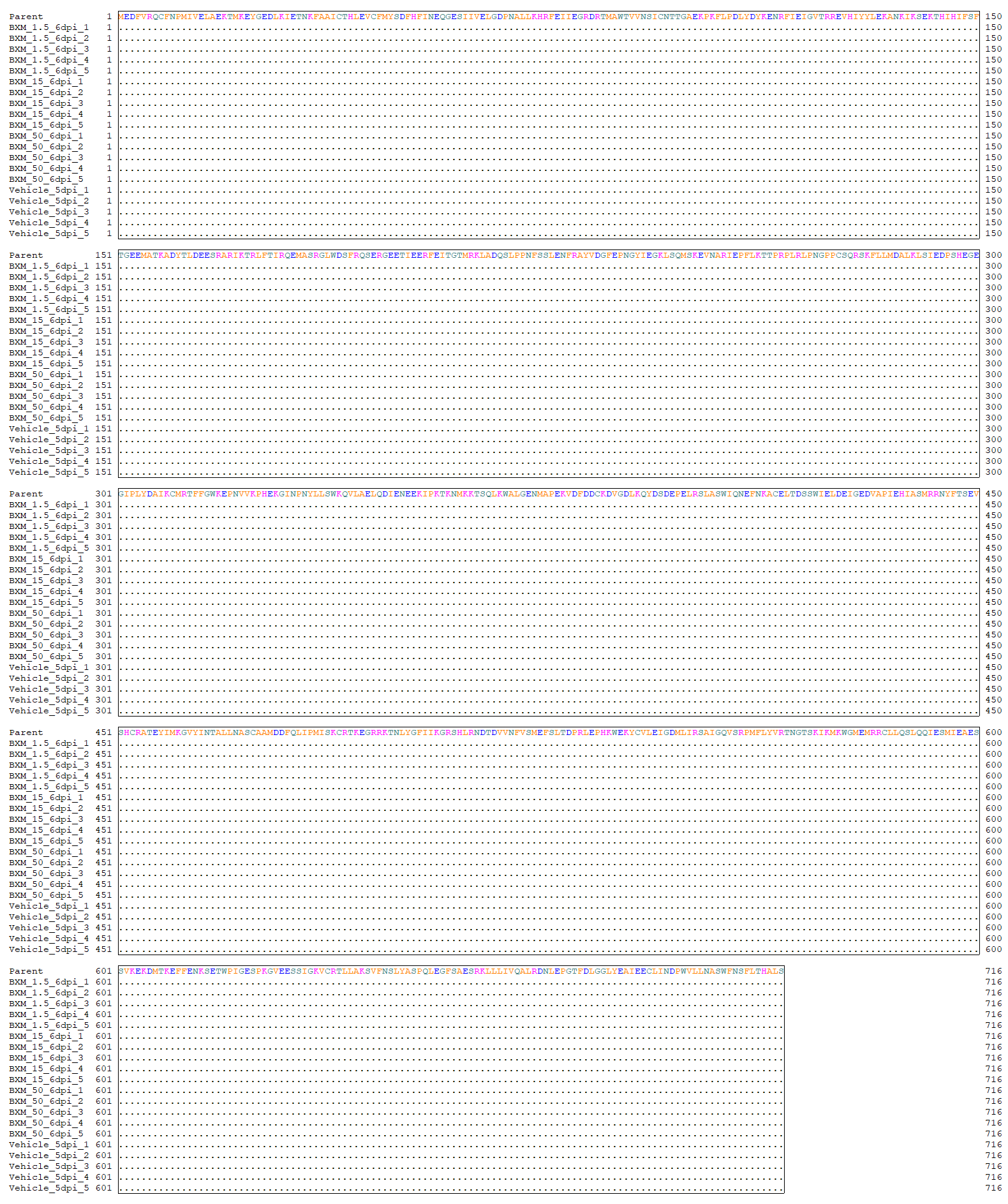

Supplement: S2 Fig — Sanger sequence analysis of the PA region of A/PR/8/34 strain was performed. Sample RNA derived from vehicle-treated group (sampling on 5 days p.i.), treatment groups with BXM (sampling on 6 days p.i.), and the parent virus (A/PR/8/34 strain) were subject to this analysis. Dot plot indicates that the amino acid sequence of virus derived from the treatment group is identical to that of the parent virus. (TIF) [file pone.0217307.s009.tif]

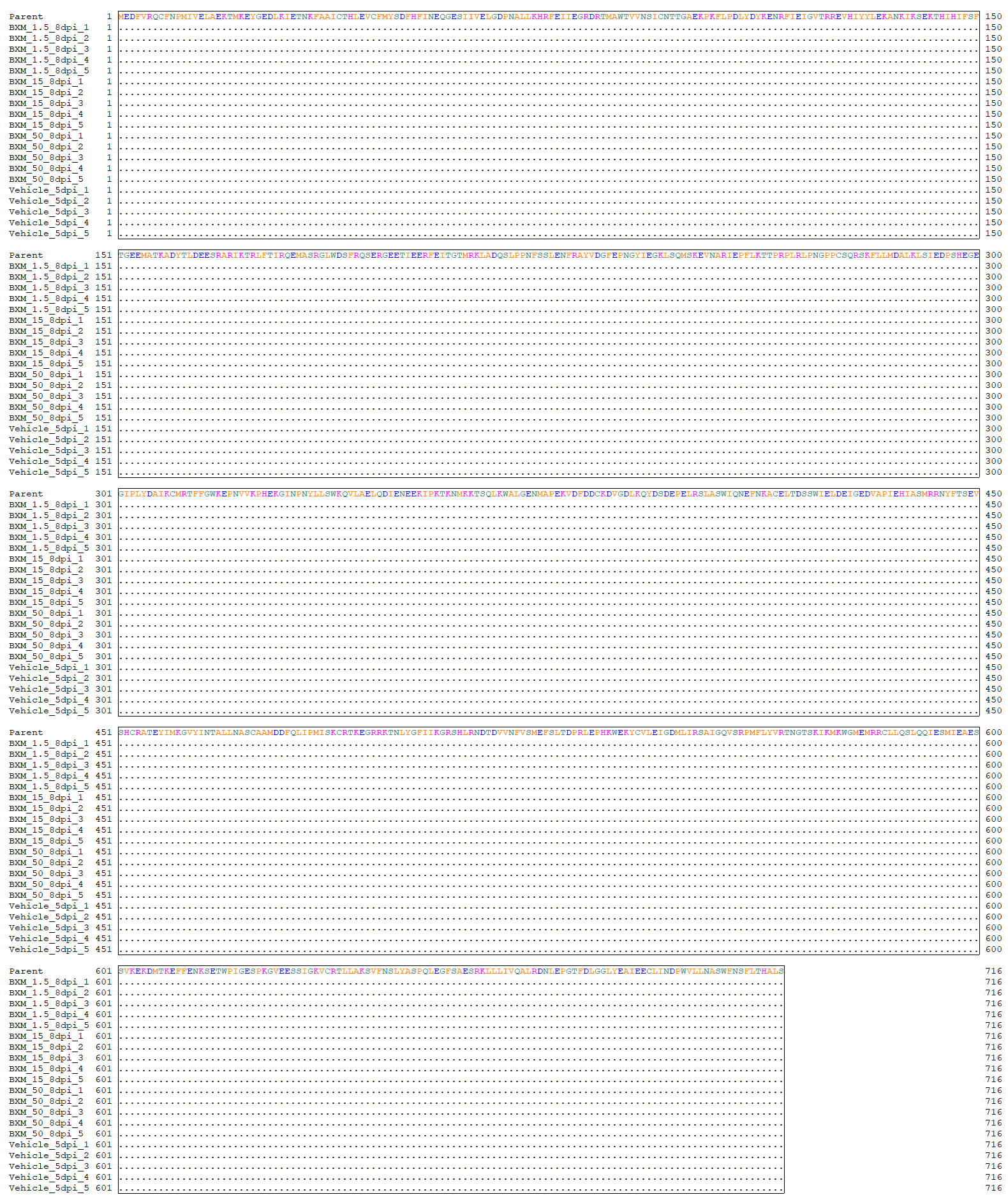

Supplement: S3 Fig — Sanger sequence analysis of the PA region of A/PR/8/34 strain was performed. Sample RNA derived from vehicle-treated group (sampling on 5 days p.i.), treatment groups with BXM (sampling on 8 days p.i.), and the parent virus (A/PR/8/34 strain) were subject to this analysis. Dot plot indicates that the amino acid sequence of virus derived from the treatment group is identical to that of the parent virus. (TIF) [file pone.0217307.s010.tif]

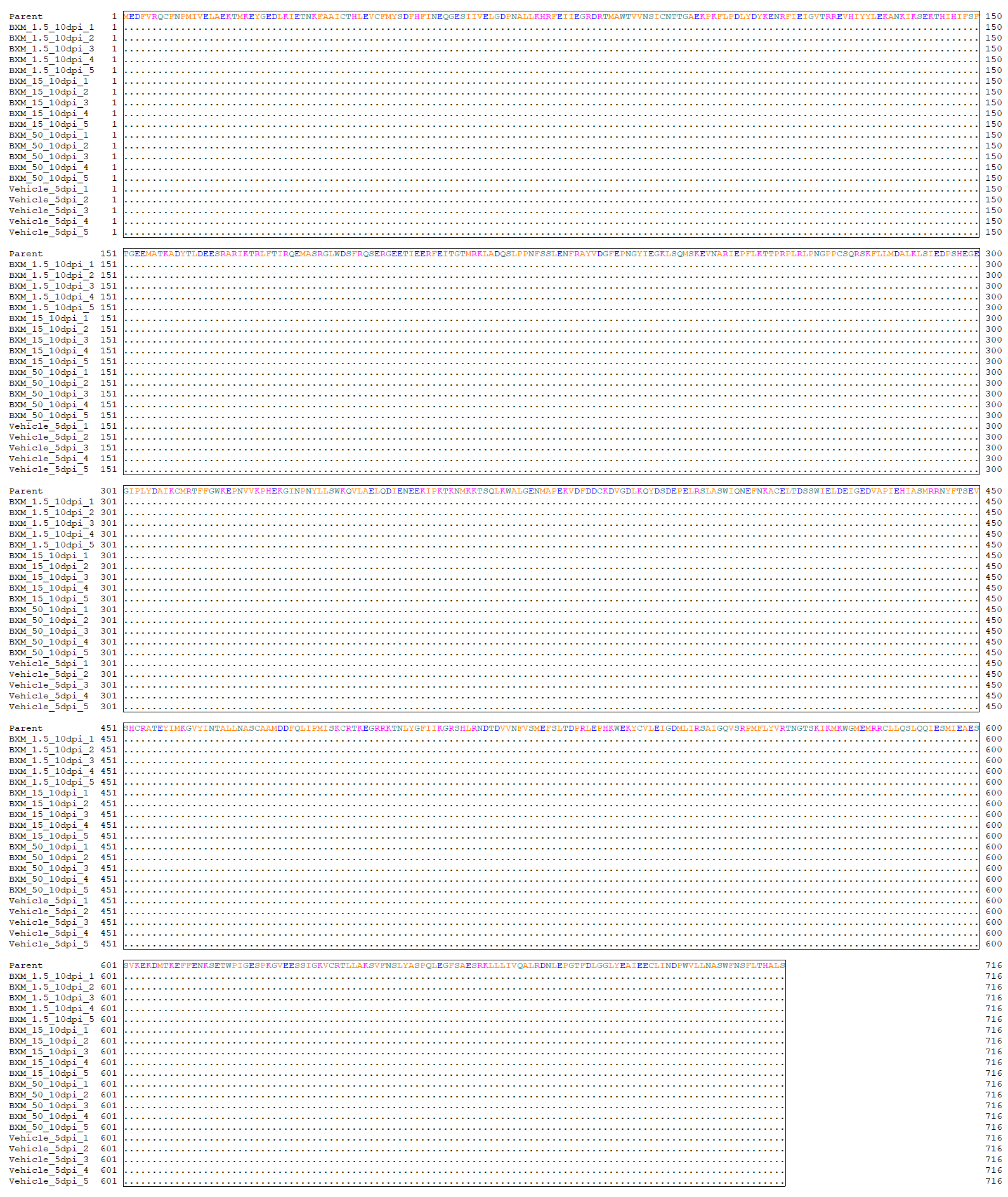

Supplement: S4 Fig — Sanger sequence analysis of the PA region of A/PR/8/34 strain was performed. Sample RNA derived from vehicle-treated group (sampling on 5 days p.i.), treatment groups with BXM (sampling on 10 days p.i.), and the parent virus (A/PR/8/34 strain) were subject to this analysis. Dot plot indicates that the amino acid sequence of virus derived from the treatment group is identical to that of the parent virus. (TIF) [file pone.0217307.s011.tif]
